# Effect of CO_2_ Laser Microperforation Pretreatment on the Dehydration of Apple Slices during Refractive Window Drying

**DOI:** 10.3390/foods12112187

**Published:** 2023-05-29

**Authors:** Helena Núñez, Aldonza Jaques, Karyn Belmonte, Andrés Córdova, German Lafuente, Cristian Ramírez

**Affiliations:** 1 Departamento de Ingeniería Química y Ambiental, Universidad Técnica Federico Santa María, Valparaíso 2390123, Chile; aldonza.jaques@usm.cl (A.J.); karyn.belmonte.12@sansano.usm.cl (K.B.); german.lafuente.14@sansano.usm.cl (G.L.); cristian.ramirez@usm.cl (C.R.); 2 Programa de Doctorado de Ciencias Agroalimentarias, Facultad de Ciencias Agronómicas y de los Alimentos, Pontifica Universidad Católica de Valparaíso, Valparaíso 2360100, Chile; andres.cordova@pucv.cl; 3 Escuela de Alimentos, Pontificia Universidad Católica de Valparaíso, Valparaíso 2360100, Chile

**Keywords:** CO_2_ laser microperforation, refractive window drying, bioactive compounds

## Abstract

This research studied the use of CO_2_ LASER microperforation as a pretreatment for the refractive window (RW) drying of apple slices with respect to total polyphenol content (TPC), antioxidant capacity, color Δ*E*, and product stability under accelerated storage. For this purpose, the processing variables assessed were pore size (200–600 µm), pore density (9–25 pores/cm^2^), and drying temperature (70–90 °C). As baseline criteria, a comparison with respect to the control without microperforations and samples subjected to conventional tunnel and lyophilization were also considered. The increase in the pore size from 200 to 600 µm resulted in shorter drying times (≤40 min), minimal change in color (Δ*E*) and loss of TPC, while DPPH was negatively affected by the combined effect of the pore density and the drying temperature. In general, the use of RW with CO_2_ resulted in apples of higher quality than those obtained in conventional drying and comparable to those obtained through the use of freeze-drying. Finally, during accelerated storage, quality attributes decreased significantly for samples dried at 90 °C regardless of whether microperforations were used, suggesting that a compromise between drying temperature and pore size must be weighed to reduce processing time and to avoid further quality losses during storage.

## 1. Introduction

Fruits provide basic nutrition along with significant health benefits for humans, but many of these fruits are produced seasonally and therefore may not be available to consumers year-round. For this reason, dried fruits represent an opportunity for consumers, since by eliminating the greatest amount of water content through various drying techniques, storage and transportation costs are reduced, and their shelf lives are increased because their stability to chemical reactions and microbial activity is improved [1]. Since the application of high temperatures during drying adversely affects the quality of dry products, it is important to select the appropriate technology [2]. Currently, the most common process of industrial food drying is convection drying or conventional drying [3]. These drying methods present long drying times and low thermal efficiency, which in turn results in lower quality products [1]. On the other hand, freeze-drying uses low-temperature operation [4], and it does not produce degradation of the food components due to thermal exposure, so nutritional reduction or sensory degradation is minimized [5]; however, it presents high capital and operational costs. The refractive window (RW) process is a fourth-generation drying technology in which hot water is used as the heating medium and circulates through the reservoir on a flexible polyester film (e.g., Mylar), where food material (pulp, juice, or sliced food) is placed. In this process, drying takes place by means of conduction and radiation, as the thermal energy is transferred from hot water to the food material through the film. Furthermore, moisture removed from the food material in the form of water vapor is carried away by a flow of air [6]. This technology has high thermal efficiency and higher heat and mass transfer rates [1,6,7] and decreased drying times, which has a positive impact on the retention of bioactive compounds and sensory properties and results in high retention of product quality [2,8,9].

Regardless of the drying method, the reduction in operating times is key to improving the productivity of any industry, which is why several technologies have been studied to improve mass and heat transfer, such as vacuum impregnation [10,11,12,13], pulsed electric fields [14,15,16,17], and ultimately, CO_2_ laser microperforation [18,19,20,21,22,23,24,25].

The CO_2_ laser is a device that generates a monochromatic, coherent, and directional beam of light that allows small holes to be made along a surface, thus causing minimal damage, without mechanical contact with the material [26]. This technology is an interesting proposal for the food industry since it allows a wavelength, whose energy is absorbed in large quantities by water, does not generate cross-contamination and allows an increase in the mass and heat transfer area during drying without damaging adjacent areas where it is applied [21]. Araya et al. [18] studied the effect of CO_2_ laser microperforation pretreatment on the dehydration of apple slices at 70 °C in drying techniques with an osmotic dehydration pretreatment. Promising results were obtained by significantly reducing the drying time compared to the control treatment (conventional drying) by more than 50%; however, the effect of microperforation pretreatment and different drying temperatures on bioactive compounds and their antioxidant capacities was not studied. Chen et al. [27] studied the effect of CO_2_ laser perforation as a pretreatment for blueberry skin before infrared freeze-drying. Laser perforation effectively increased drying speed, helped reduce shrinkage, increased rehydration capacity, and enhanced total phenolic compounds in dehydrated fruits.

The objective of this research is to determine the effect of microperforations with CO_2_ laser technology as a pretreatment in the drying of apple slices (Granny smith) in refractive window technology in terms of total polyphenols, antioxidant capacity, color, drying times, and product stability under accelerated storage. As baseline criteria, comparison with conventional tunnel drying and lyophilization has also been considered. The study was performed using Granny smith apple slices as a model food, considering tissue and maturity homogeneity.

## 2. Materials and Methods

### 2.1. Materials

Granny Smith (GS) apples (*Malus domestica*) were purchased from a local supermarket in Valparaíso, Chile, and they were refrigerated at 2 ± 0.5 °C until use. Apples with 11 ± 1° Brix and slices were obtained exclusively from the parenchymatic tissue based on the work ok Ramírez et al. [28]. A solution with 2% and 1% citric and ascorbic acid, respectively, was prepared to avoid enzymatic browning of the apple samples after microperforations. All of the reagents were purchased from G.A. Sales. Apple slices were cut using a kitchen mandolin and cork borer to provide a sample thickness and diameter of 0.004 ± 0.0001 m thickness and 0.040 ± 0.001 m diameter, respectively, and each slice had an average weight of 0.003 ± 0.00048 kg.

### 2.2. Experimental Design

**Figure 1:**
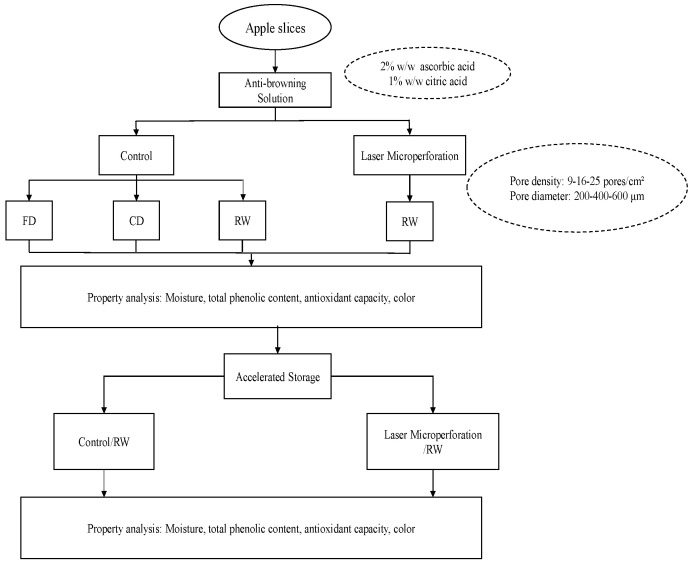
Experimental design.

To determine the effect of microperforations on the total polyphenols, antioxidant capacity, and color as response variables, an experimental design was carried out. The variables were the operating temperature of the RW (from 70 to 90 °C), the pore diameter (from 200 to 600 µm), and the pore density (9 to 25 pores/cm^2^) in the slice. The experimental variables were combined in a Box–Behnken design (BBD). RW drying at 70, 80, and 90 °C without microperforations was used as a control experiment. In addition, hot air drying in a tunnel at 70 °C and lyophilization were used for comparison to commercial existing drying processes. Figure 1 presents the proposed experimental design.

#### 2.2.1. CO_2_ Laser Microperforations

The laser microperforations (LMP) were applied to the samples using a SYNRAD TI100 CO_2_ laser (Firestar t100, Synrad Inc. Mukilteo, WA, USA) with a 125 nm lens, which was linked to the computer with WinMarkPro Laser Making Software, allowing for its configuration and adjustment. The parameters of residence time, number of pulses and power were established to pass through 100% of the sample, according to Araya et al. [18] (Table 1). The pore density (PD, the number of pores per unit area) configuration was conducted in honeycomb arrangements using 9, 16, and 25 pores/cm^2^. The pore size generated was measured by optical microscopy (H 600 LL HP 100, Hund Wetzlar, Wetzlar, Germany) using the uEye Cockpit software from Imaging Development Systems 113 (IDS) and Image-Pro Plus, which allows for capturing a photo of a microscopic observation [20].

#### 2.2.2. Conventional Drying (CD)

The conventional drying equipment (CD) used was a hot-air drying tunnel that was 2.7 m long and 0.4 m wide (15 KW, 80 V). On one side, there is a fan that drives the air at an average speed of 1.53 ± 0.25 m/s. The equipment capacity was 48 samples per drying process, which were placed on a support that was in the middle of the tunnel. The drying temperature was 70 °C for 135 min (the water activity attained for the dried samples was 0.316 ± 0.026).

#### 2.2.3. Refractance Window^TM^ (RW^TM^)

The refractive window was made up of a thermoregulated water bath (Mermmet, model WND), in which a Mylar^®^ sheet (thicknesses 0.1 mm) measuring 0.3 m wide by 0.5 m long was placed over the water, as according to Hernández et al. [29]. Sixty apple slices were placed on the Mylar sheet. The relative humidity in the laboratory was 47 ± 8%. The process ended when the samples reached a water activity (a_w_) below 0.4. At a_w_ < 0.4, foods do not show microorganism spoilage [30]; furthermore, apple browning is reduced [31].

#### 2.2.4. Freeze-Drying (FD)

All experiments were carried out in a Martin Christ freeze-dryer, model Alpha 2-4 LSCplus (Martin Christ Gefriertrocknungsanlagen, Osterode, Germany), which can operate at a total vacuum pressure of up to 0.3 mbar, provided with an MKS Baratron 622 capacitance manometer (MKS Instruments) and a condenser that can operate at temperatures as low as −85 °C. The apple slices were frozen in the freezer (Haier ULT Freezer, model DW-86W100J, China) at −85 °C for 24 h before being freeze-dried. The apple samples were placed onto the three shelves, considering a total load of 18 units. The drying temperature in the freeze dryer was 30 °C.

### 2.3. Determination of Moisture Content and Water Activity

The moisture content was determined by drying 5 g of the sample under vacuum conditions in an oven at 70 °C for 24 h and recording the final constant weight (AOAC method no. 934.06) [32]. The moisture content was calculated using Equation (1), where *X^W^* corresponds to the mass fraction of water (g water/g sample), and *m*_0_ and *m_f_* are the masses of the initial and dry samples, respectively (g).

(1) $$X^{W}=\frac{m_{0}-m_{f}}{m_{0}}$$

Water activity was determined using a dew-point hygrometer Aqualab series 4 TE (Decagon Devices, Inc., Pullman, Washington, DC, USA) with a resolution of 0.0001 a_w_.

### 2.4. Determination of Color

The color of the samples was evaluated using a colorimeter (model CR-410, Konica Minolta, Tokyo, Japan) to measure the coordinates of the CIEL*a*b* uniform color space, where L* is the luminosity, a* is the red–green hue, and b* is the yellow–blue hue. The type of illuminant is D65, and the degrees of the observer is 2°. The color difference was calculated using Equation (2), where Δ*E* indicates the magnitude of the color change between the initial (*L*_0_*, *a*_0_* y *b*_0_*) and final (L_f_*, a_f_* y b_f_*) parameters of the sample to be compared [33]. Δ*E* was obtained for all cases using a fresh apple and the dried controls for comparison with the dried samples.

(2) $$∆E=\sqrt{{∆L}^{*2}+{∆a}^{*2}+{∆b}^{*2}}$$

### 2.5. Determination of Total Polyphenol Content (TPC) and Antioxidant Activity

The extraction of polyphenols was performed by extracting two grams of the ground sample (fresh or dried) with 20 mL of an 80% methanol solution using a homogenizer at room temperature and protected from light for 1 h. The supernatant was filtered through Whatman paper No. 2 and stored at −20 °C.

The total polyphenol content in the extracts was determined using the Folin–Ciocalteu method [34] and expressed as gallic acid equivalents (GAEs) in mg/g dry matter.

The antioxidant capacity was determined using DPPH (2,2-diphenyl-2-picryl-hydrazyl) as performed by Galaz et al. [34]. The results were expressed as Trolox equivalents in μmol/g dry matter.

The total polyphenol content and antioxidant capacity were obtained using a Genesys 5 spectrophotometer (Spectronic Instrument, Inc., model 336001, New York, NY, USA) by measuring the absorbance at 765 and 517 nm, respectively.

### 2.6. Accelerated Storage Test

Accelerated storage tests were carried out in an oven with humidity control (Memmert, model HCP-108). The dehydrated apples were kept in aluminum Ziploc bags (11 × 16 × 3 m). The packaged samples were stored at 45 °C under constant accelerated relative humidity (75%) conditions for 4 weeks. The samples were analyzed for selected quality attributes, namely, antioxidant capacity (AC) (μmol/g dry matter), total phenolic content (TPC) (mg gallic acid equivalents (GAE)/g dry matter), moisture content (MC) (g water/g sample), and total color change (Δ*E*).

### 2.7. Experimental Design and Statistical Analysis

All of the data were reported as the means of three replicas and their respective standard deviations. The effect of microperforations on the RW was analyzed using a response surface methodology based on a Box–Behnken design (BBD) with three center points, Table 2 shows the values of each variable with their respective level in the proposed experimental design. The significance test of the results was performed with an analysis of variance (ANOVA) and Duncan’s multiple range tests with a significance of 95% using the STATGRAPHIC Centurion XVIII software, Statpoint Inc.^®^, 2018.

## 3. Results and Discussion

### 3.1. Color

Color is an important attribute that plays a significant role in food acceptance [35]. Table 3 shows the measured color parameters of fresh and dried apple slices. The fresh apple slice had a value of L* = 74.77 ± 1.17, a* = −5.47 ± 0.70 and b* = 19.69 ± 2.09, similar to reports from Araya et al. [18], Henríquez et al. [36] and Hernández et al. [29].

With respect to delta color, comparing the results obtained between the control treatments, the value Δ*E* for the RW technique is slightly lower than that obtained for conventional drying; nevertheless, there are no significant differences between the different temperatures (*p* < 0.05). Similar results were obtained by other researchers, Franco et al. [37] and Hernández et al. [29]. In addition, when compared with the freeze-drying process, no significant differences were found (*p* < 0.05), similar to Caparino et al. [38] in mango and Puente et al. [39] in goldenberry. In the cases without microperforations, Δ*E* was between 11.26 and 12.56, and for the treatments with microperforations, Δ*E* presented values between 5.89 and 11.35.

The Δ*E* values were, in most cases, significantly lower when laser microperforations were applied in comparison to the control samples. The smallest color differences (Δ*E* = 5.89) were obtained in treatments 2 and 3 (17 pores/cm^2^, 600 µm) at 70 and 90 °C, respectively. In general, microperforation pretreatments resulted in products that indicated lower degradation of the color, probably due to a reduction in process time with respect to the control. Table 4 shows the drying time; for the three temperatures, a decrease in drying times was observed, and the case of 70 °C, the application of different combinations of pore densities with different pore diameters presented a decrease of approximately 20% in the processing time. At 80 °C, the reduction in drying time was between 5 and 25% compared to the control treatment, while at 90 °C, the reduction values were between 2 and 13%, and the degradation of the product’s original color appeared after long drying time exposure [37,40,41]. In general, there are no significant differences between the treatments with microperforations; however, it is possible to obtain a product with a similar delta color but with less processing time.

### 3.2. Total Phenolic Content (TPC)

**Figure 2:**
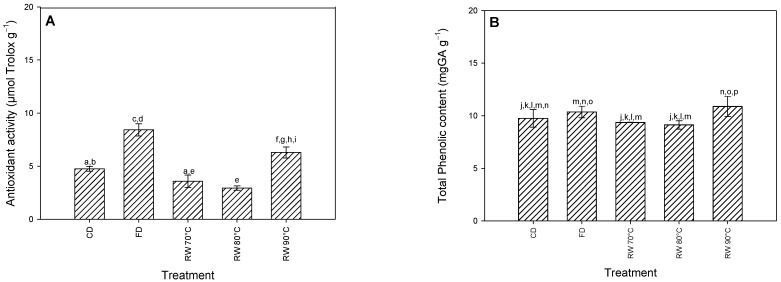
Effect of drying on (**A**) antioxidant activity and (**B**) total phenolic content (CD: conventional drying; FD: freeze drying; RW: refractance window). a–i: Different lowercase letters show that the results are statistically significantly different (*p* < 0.05) for antioxidant activity. j–p: Different lowercase letters show that the results are statistically significantly different (*p* < 0.05) for total phenolic content.

The fresh raw material had a total polyphenol content of 16.79 ± 0.56 mg GAE/g_bs_ (2.25 mg GAE/g_bh_) with similar results to those reported in Mitić et al. [42] in Granny smith apples (1.97 mg GAE/g_bh_). Figure 2A shows the results of the total polyphenol content obtained from the control experiments. There were no significant differences (*p* < 0.05) between the freeze-drying process and the RW at 90 °C. The greater retention of TPC in dehydrated samples at higher temperatures (90 °C) could be explained by the significant decrease in drying time (45 min), which decreases the thermal and oxidative degradation of phenolic compounds [43]. As already mentioned, during conventional drying at 70 °C, the drying time took place in 135 min, but despite the use of RW at 70 °C and 80 °C reducing the drying time to 120 and 80 min, respectively, such decreases were not enough to prevent spoilage of the bioactive compounds, as they presented similar concentrations (*p* ≤ 0.05). The same trend has been reported through a comparison of RW at different temperatures with conventional drying of banana puree [44]. The difference in processing time can be explained by the drying mechanisms; the main process of freeze-drying is the sublimation and subsequent diffusion of water within the product in a vacuum environment; however, it requires a large consumption of energy and time. In the case of conventional drying (conduction and convection), they cause a decrease in the quality of the product in terms of nutritional, color, and functional properties due to the drying temperatures and processing times [45], while the RW drying can be performed at higher temperatures, because during the RW drying process, the three heat transfer mechanisms occur: conduction, convection and radiation. As the product loses moisture, the “window” closes and begins to “refract” the infrared radiation towards the water source, increasing the reflextivity [46]. At this stage, heat transfer through conduction is predominant, and evaporation is maintained until reaching the critical moisture content of the product. In the last stages of drying, a reduction in heat transfer occurs, which protects the product from overheating, thanks to the effects of evaporative cooling [47].

**Figure 3:**
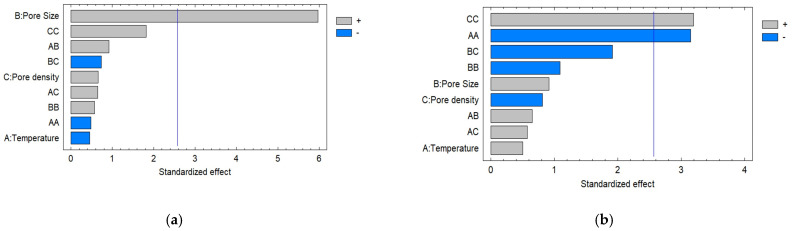
Standardized Pareto charts (α = 0.05) of effects of pore size, pore density, and temperature for (**a**) TPC and (**b**) antioxidant capacity.

**Figure 4:**
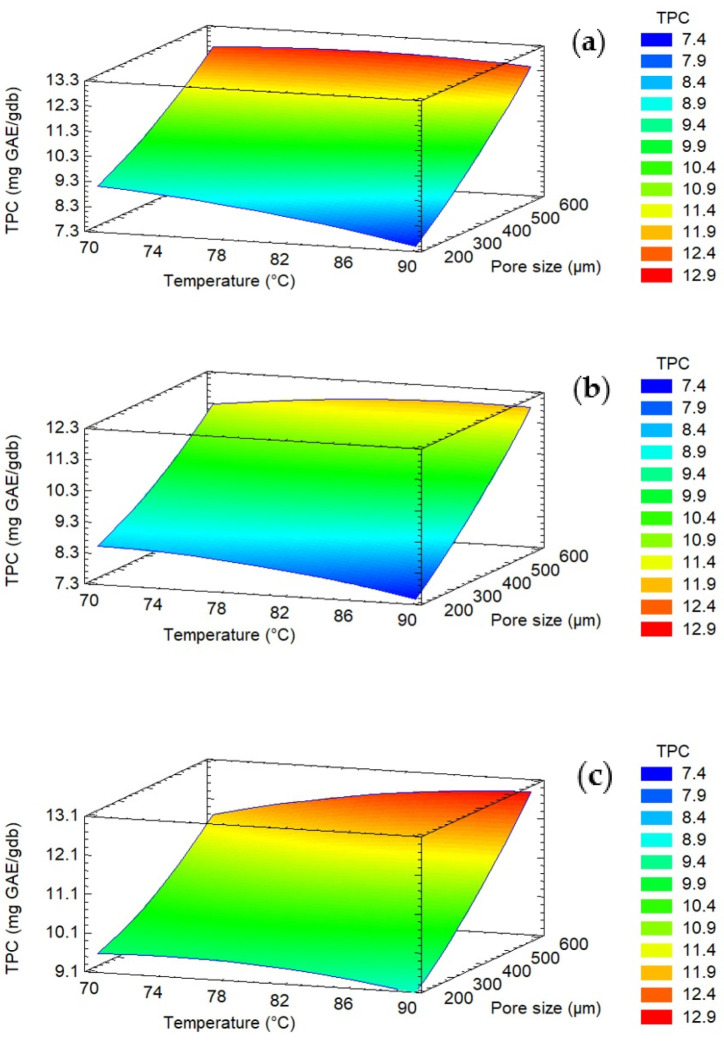
Surface response of the effects of pore size, pore density, and temperature on the TFC. (**a**) pore density = 9 pores/cm^2^, (**b**) pore density = 17 pores/cm^2^, (**c**) pore density = 25 pores/cm^2^.

The results show that the minimum value corresponds to Experiment 1 (17 pores/cm^2^, 200 µm, 90 °C), 6.59 ± 0.25 mg GAE/gbs, and the maximum TPC retention was in the case of Experiment 12 (9 pores/cm2, 600 µm, 80 °C), 12.49 ± 0.41 mg GAE/gbs. When comparing the control treatments and those with microperforations, significant differences in TPC were observed when a larger pore size (600 µm) was used for the cases of 70 and 80 °C, obtaining higher values in the case of the treatments with microperforations. In this regard, the Pareto chart (Figure 3a) shows that the pore diameter is the main variable that has a significant effect on the TPC. Figure 4 shows the interrelationship between the drying temperature, pore size, and TPC content of apple fruits. The figure results clearly show that increasing the pore size promotes the retention of polyphenols, and then the TPC content increases. Table 4 shows the a_w_ values of all treatments; in general, there are no significant differences (*p* < 0.05) in the different treatments, with the exception of treatments 3, 10 and 11, which present lower values. a_w_ is a key factor affecting the stability of polyphenols [48]. Tonon et al. [49] studied the anthocyanin stability of spray-dried açai juice and found that increased water activity resulted in greater degradation. This was attributed to the higher molecular mobility, which allows easier diffusion of oxygen, thus accelerating oxidation reactions. The water content (Table 3) is one of the most influential factors in nutrient degradation. As water is extracted and the food material shrinks, the chemical concentration increases, but certain water-soluble compounds may serve as decomposition catalysts [50]. Additionally, the degradation of TPC is well known to be related to the duration and intensity of heating; therefore, the time–temperature regime during the process should be considered apart from the pore diameter to retain them as high as possible. The response surface methodology analysis showed that the lack of fit was not significant for all response variables (*p* > 0.05), and the R-squared statistic for the response surface model was 90.75, while the R^2^_adj_ value was 74.09, which indicates its ability to explain the effect of microperforations pretreatments on the retention of TPC.

### 3.3. Antioxidant Activity

The fresh raw material had an antioxidant capacity of 15.08 ± 0.73 µmol Trolox/gbs. Figure 2B shows the results of the antioxidant capacity for the control experiments. In the control experiments, freeze-drying was the best method to retain the antioxidant activity with respect to the raw material, attaining a value of 8.42 ± 0.57 µmol Trolox/g_db_. The drying tunnel and the refractive window at 70 and 80 °C present the lowest antioxidant capacity of the series of experiments carried out with values of 4.75 ± 0.22, 3.58 ± 0.58, and 2.94 ± 0.52 µmol Trolox/g_db,_ respectively. On the other hand, the refractive window at 90 °C (6.29 ± 0.22 µmol Trolox/g_db_) presents a higher retention of antioxidant capacity compared to conventional drying; however, significant differences are evident with freeze-drying. Baeghbali et al. [51] reported that there are no significant differences between drying in a refractive window at 91 °C of pomegranate juice and freeze-drying. On the other hand, Nayak et al. [52] presented the results of drying flakes of various colored potatoes through different drying methods, such as a refractive window at 95 °C, drum drying with steam at 145 °C, and freeze-drying. The results obtained concerning antioxidant capacity showed that there was no significant difference between the drying methods. Galaz et al. [34] used a combination of high temperatures and short processing time during the drying of pomegranate peel using a drum dryer. The combination of temperature–time allowed for the removal of dried products of pomegranate peel that maintain their antioxidant activity. Therefore, the time–temperature relationship is a determining factor in the retention of the antioxidant capacity. 

Laser microperforation allowed antioxidant capacity to be obtained in several treatments without significant differences in comparison with the freeze-drying process (*p* > 0.05). For example, at 70 °C, treatment 8 (9 pores/cm^2^, 400 µm) was 7.21 ± 0.26 µmol Trolox/gdb; at 80 °C, treatments 11 (25 pores/cm^2^, 600 µm) and 12 (9 pores/cm^2^, 600 µm) were 7.68 ± 1.98 µmol Trolox/gdb and 8.87 ± 0.21 µmol Trolox/gdb, respectively, and at 90 °C, treatment 6 (25 pores/cm^2^, 400 µm) was 7.15 ± 1.02 µmol Trolox/gdb. These results suggest that the combination of drying temperature, pore size, and pore density favors the retention of antioxidant capacity, with values higher than those of the control treatments and not presenting significant differences with freeze drying.

**Figure 5:**
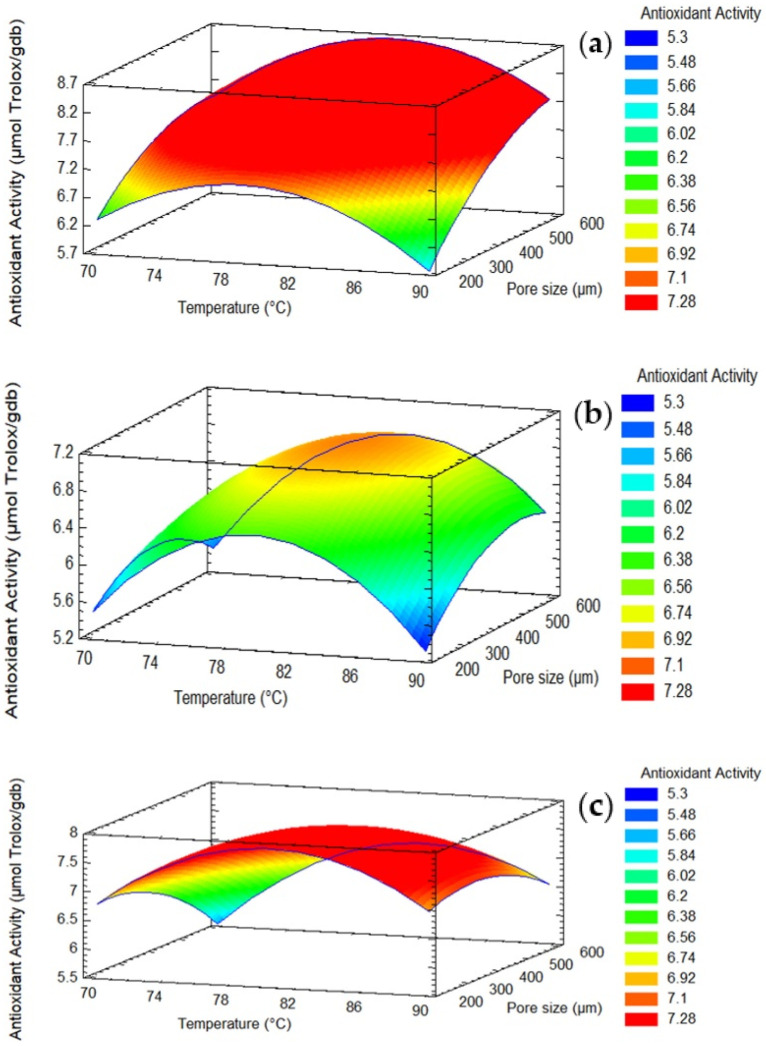
Surface response of the effects of pore size, pore density and temperature on the antioxidant activity. (**a**) pore density = 9 pores/cm^2^, (**b**) pore density = 17 pores/cm^2^, (**c**) pore density = 25 pores/cm^2^.

According to the results (Figure 3b), the factors with the largest effects on antioxidant activity were the quadratic effects terms temperature and pore density. Figure 5 presents the surface plot for the interaction effect between the pore size and temperature on the antioxidant capacity. The results showed that the maximum antioxidant capacity was attained at a temperature of 80 °C, a range of pore sizes larger than 400 μm, and a density of 9 pores/cm^2^ (Figure 5).

### 3.4. Accelerated Storage Test

The accelerated storage tests apply the principles of chemical kinetics to quantify the effects that extrinsic factors such as temperature, humidity, and light have on the rate of deterioration reactions. By exposing food to controlled environments in which one or more of the extrinsic factors are held at a higher-than-normal level, spoilage rates are accelerated, resulting in a shorter-than-normal time for the product to fail [53]. Although the use of microperforations tended to retain the concentration of TPC in comparison to the control treatments, it is interesting to evaluate the effect on these compounds during storage. Hence, an accelerated storage study was carried out. The quality parameters and bioactive properties of the control cases (RW at 70, 80 and 90 °C) and of the two best experiments with perforation were analyzed according to the results obtained for color difference, antioxidant activity, TPC, and moisture (Table 5). The accelerated storage study was carried out for 4 weeks, comparing the results at the beginning and end of the period. Based on the results, Experiment 2 was chosen because it presents the lowest value of color difference. In addition, Experiment 6 was chosen because it presents one of the highest antioxidant capacity profiles and total polyphenol content among the series of experiments.

The results show a significant increase in the moisture content (*p* < 0.05) between weeks 0 and 4. Although the packaging may serve as a protective material against food spoilage, there will always be gas and water permeability, which are increased at high storage temperatures, such as in the current case, but other factors, such as pH, oxygen, material porosity and light, may also be affected [54]. In fact, it has been observed that dried apples can be stable and result in a good source of antioxidant compounds as long as packing materials with a high barrier against moisture sorption are used, resulting in the latter being the most detrimental factor [55,56]. The results from Table 5 also show that TPC degradation increased with increasing drying temperature (*p* < 0.05). For instance, at 70 °C and 80 °C, TPC retention was on average ~84%, while at 80 °C and 90 °C, TPC retention was ~72% regardless of whether microperforation pretreatments were performed. Regarding the antioxidant activity values of apple slices, no significant changes were observed after storage for controls at 70 °C and 80 °C; however, the increase in drying temperature at 90 °C resulted in ~42% retention regardless of whether microperforation pretreatment was used or not. This situation may be explained because the use of higher temperatures during processing may result in further autocatalytic reactions, especially in products subjected to concentrations where there is also the presence of sugars and reduced a_w_ [57]. The color difference (ΔE*) between the dried apple slices at weeks 0 and 4 was determined. The results (Table 5) show that there were significant differences (*p* < 0.05) between the cases studied, except for the control at 90 °C in Experiment 6. At the end of the accelerated storage, the samples present high values of ΔE*, ranging between 26.29 and 37.11, with Experiment 2 being the one that obtained the least color variation. The control samples present a lower color change ratio, which may be because the matrix without perforations protects from the reactions produced by the enzymes, the presence of water and the temperature during storage, avoiding, in the short term, more significant browning than in the perforated samples. Another observation is that the control samples, at a higher drying temperature, produce a lower color difference, but in storage, the samples dried at 90 °C show a greater color change than the samples at other temperatures. This may be because the samples dried at 90 °C present a greater retention of the bioactive components, which are more likely to react with ambient conditions and to reactivate enzymes (for example, polyphenol oxidase).

Regarding the perforated samples, an increase in the color difference greater than the control samples was observed. This behavior could be explained due to the presence of micropores that allow to increase the surface area in which bioactive compounds can react with oxygen. At 4 weeks, all samples exposed to the assay reached a similar order of magnitude in color difference, although a greater difference was observed in samples that were perforated.

## 4. Conclusions

For apple slices dried using RW, the application of laser microperforation allowed for the color retention, total polyphenol content, and antioxidant capacity to be comparable to those obtained through the freeze-drying process. Regarding the drying time required to get an a_w_ lower than 0.4, it was significantly lower in microperforated samples, attaining a time reduction in the range of 2–25% (which depends on drying temperature) compared with non-perforated samples. The pore size is a relevant variable in the retention of bioactive compounds, obtaining higher polyphenols content as pore size increased. In accelerated storage, the microperforated samples presented a higher loss of bioactive compounds, which could be explained due to an increase in the mass transfer area due to the presence of pores. However, an adequate design of pore size and distribution must be performed in order to create a pore that, during drying, remains open, allowing water migration, but, as drying occurs, closes. Therefore, this would avoid the loss of bioactive compounds due to increases in mass transfer area during drying.

## Figures and Tables

**Table 1:** Different configurations of power, residence time, and number of pulses were used to obtain the three pore sizes (PS) tested.

		Pore Size (PS)	
Parameter	PS1 203.25 ± 23.05 μm	PS2 417.89 ± 6.14 μm	PS3 623.41 ± 7.50 μm
Power (%) (based on 100 W)	10	25	75
Residence Time (μs)	2	1	1
Number of pulses	120	120	120

**Table 2:** Box–Behnken design for surface analysis with 15 treatments in total, including 3 central points.

Experiments	Temperature	Pore Size	Pore Density
1	1	−1	0
2	1	1	0
3	−1	1	0
4	−1	−1	0
5	1	0	−1
6	1	0	1
7	−1	0	1
8	−1	0	−1
9	0	−1	−1
10	0	−1	1
11	0	1	1
12	0	1	−1
13	0	0	0
14	0	0	0
15	0	0	0
FACTORS	−1	0	+1
$T_{i}$ (°C)	70	80	90
dpi (µm)	200	400	600
Dpi (pores/cm^2^)	9	17	25

Where *T_i_*, *dpi* and *Dpi* correspond to the variables of temperature, pore diameter and pore density, respectively, for the corresponding level *i*.

**Table 3:** Mean values of moisture and color parameters for fresh apple and all treatments dried.

	Experiment	Moisture (g Water/g Sample)	Δ*E*
	Fresh	0.866 ± 0.002 ^a^	
	CD	0.023 ± 0.002 ^b^	13.26 ± 0.15 ^a^
	FD	0.011 ± 0.001 ^c^	11.77 ± 0.05 ^b^
70 °C	Control RW	0.038 ± 0.001 ^d.e^	12.06 ± 0.17 ^a,b^
	3 (17 pores/cm^2^, 600 µm)	0.044 ± 0.001 ^d,e,f^	5.89 ± 0.39 ^c^
	4 (17 pores/cm^2^, 200 µm)	0.103 ± 0.011 ^g,h^	11.35 ± 1.11 ^b,d^
	7 (25 pores/cm^2^, 400 µm)	0.063 ± 0.007 ^i^	6.92 ± 0.50 ^c.j^
	8 (9 pores/cm^2^, 400 µm)	0.096 ± 0.001 ^g^	11.09 ± 0.66 ^b,k^
80 °C	Control RW	0.035 ± 0.002 ^d^	11.26 ± 1.26 ^b^
	9 (9 pores/cm^2^, 200 µm)	0.014 ± 0.001 ^b,c^	8.90 ± 1.21 ^e,f,i^
	10 (25 pores/cm^2^, 200 µm)	0.094 ± 0.015 ^g^	8.75 ± 0.44 ^f,i^
	11 (25 pores/cm^2^, 600 µm)	0.055 ± 0.002 ^f,i^	8.22 ± 1.46 ^e,f,g,i^
	12 (9 pores/cm^2^, 600 µm)	0.057 ± 0.002 ^i^	8.98 ± 0.90 ^e,f,i^
	13 (17 pores/cm^2^, 400 µm)	0.097 ± 0.007 ^g^	8.48 ± 1.21 ^e,f,h,i^
	14 (17 pores/cm^2^, 400 µm)	0.067 ± 0.006 ^i^	8.83 ± 1.04 ^e,f,i^
	15 (17 pores/cm^2^, 400 µm)	0.055 ± 0.003 ^f,i^	9.65 ± 0.94 ^e,f^
90 °C	Control RW	0.049 ± 0.001 ^e,f^	12.56 ± 0.91 ^a,b,d^
	1 (17 pores/cm^2^, 200 µm)	0.065 ± 0.002 ^i^	7.19 ± 0.89 ^c,g,h,i^
	2 (17 pores/cm^2^, 600 µm)	0.048 ± 0.002 ^e,f^	5.89 ± 1.62 ^c^
	5 (9 pores/cm^2^, 400 µm)	0.092 ± 0.007 ^g^	9.63 ± 0.07 ^f,k^
	6 (25 pores/cm^2^, 400 µm)	0.111 ± 0.005 ^h^	7.76 ± 0.04 ^i,j^

Means with different superscripts within a column differ significantly (*p* < 0.05).

**Table 4:** Mean values (with standard deviation) of water activity (a_w_).

	Experiment Refractance Window	a_w_	Time (min)
70 °C	Control	0.361 ± 0.027 ^a,b,c^	120 ± 6.4 ^a^
	3 (17 pores/cm^2^, 600 µm)	0.286 ± 0.019 ^d,e^	93 ± 9.2 ^b^
	4 (17 pores/cm^2^, 200 µm)	0.357 ± 0.015 ^a,b,c^	90 ± 0.71 ^b^
	7 (25 pores/cm^2^, 400 µm)	0.321 ± 0.031 ^a,d,e^	90 ± 6.4 ^b^
	8 (9 pores/cm^2^, 400 µm)	0.388 ± 0.012 ^b,c^	95 ± 1.4 ^b^
80 °C	Control	0.376 ± 0.009 ^a,b,c^	80 ± 4.2 ^c^
	9 (9 pores/cm^2^, 200 µm)	0.361 ± 0.027 ^a,b,c^	76 ± 2.8 ^c,d^
	10 (25 pores/cm^2^, 200 µm)	0.222 ± 0.024 ^f^	70 ± 4.2 ^d,e^
	11 (25 pores/cm^2^, 600 µm)	0.264 ± 0.022 ^d,f^	60 ± 4.9 ^f^
	12 (9 pores/cm^2^, 600 µm)	0.361 ± 0.033 ^a,b,c^	63 ± 3.5 ^e,f^
	13-14-15 (17 pores/cm^2^, 400 µm)	0.342 ± 0.060 ^a,b,e^	60 ± 4.2 ^f^
90 °C	Control	0.360 ± 0.033 ^a,b,c^	45 ± 3.5 ^g^
	1 (17 pores/cm^2^, 200 µm)	0.358 ± 0.029 ^a,b,c^	43 ± 2.1 ^h^
	2 (17 pores/cm^2^, 600 µm)	0.378 ± 0.016 ^a,b,c^	37 ± 1.4 ^h^
	5 (9 pores/cm^2^, 400 µm)	0.353 ± 0.026 ^a,b,c^	44 ± 2.8 ^h^
	6 (25 pores/cm^2^, 400 µm)	0.408 ± 0.014 ^c^	39 ± 2.1 ^h^

Means with different superscripts within a column differ significantly (*p* < 0.05).

**Table 5:** Average values of moisture, Δ*E*, TPC and antioxidant activity for samples stored over time.

	Experiment	Moisture (g Water/g Sample)	Δ*E*	TPC	Antioxidant Capacity
	Control 70 °C	0.038 ± 0.001 ^Aa^	-	9.365 ± 0.020 ^Aa^	3.575 ± 0.582 ^Aa^
Week 0	Control 80 °C	0.035 ± 0.002 ^Aa^	-	9.128 ± 0.403 ^Aa^	2.935 ± 0.265 ^Aa^
	Control 90 °C	0.049 ± 0.001 ^Ba^	-	10.883 ± 0.970 ^Ba^	6.292 ± 0.519 ^BCa^
	2 (17 pores/cm^2^, 600 µm)	0.048 ± 0.002 ^Ba^	-	11.672 ± 1.305 ^Ba^	7.154 ± 1.012 ^Ba^
	6 (25 pores/cm^2^, 400 µm)	0.111 ± 0.006 ^Ca^	-	11.254 ± 0.315 ^Ba^	5.494 ± 1.059 ^Ca^
	Control 70 °C	0.087 ± 0.001 ^Ab^	32.93 ± 1.21 ^A^	8.015 ± 0.190 ^ABb^	3.408 ± 0.447 ^Aa^
	Control 80 °C	0.118 ± 0.001 ^Bb^	29.54 ± 1.56 ^B^	7.587 ± 0.363 ^Ab^	3.333 ± 0.365 ^Aba^
	Control 90 °C	0.092 ± 0.001 ^Cb^	37.11 ± 2.34 ^C^	8.059 ± 0.191 ^ABb^	3.052 ± 0.763 ^Abb^
Week 4	2 (17 pores/cm^2^, 600 µm)	0.118 ± 0.001 ^Bb^	26.29 ± 0.95 ^D^	8.568 ± 0.418 ^Bb^	2.546 ± 0.087 ^BCb^
	6 (25 pores/cm^2^, 400 µm)	0.150 ± 0.001 ^Db^	36.89 ± 1.18 ^C^	7.906 ± 0.221 ^Ab^	2.256 ± 0.251 ^Cb^

A–D: Different uppercase letters within the same column and week show that the results are statistically significantly different (*p* < 0.05). a,b: Different lowercase letters within the same column and experiment show that the results are statistically significantly different (*p* < 0.05).

## Data Availability

The data presented in this study are available on request from the corresponding author.

## References

[1] Raghavi L.M., Moses J.A., Anandharamakrishnan C. (2018). Refractance window drying of foods: A review. J. Food Eng..

[2] Ochoa-Martínez C.I., Quintero P.T., Ayala A.A., Ortiz M.J. (2012). Drying characteristics of mango slices using the refractance window^TM^ technique. J. Food Eng..

[3] Zarein M., Samadi S.H., Ghobadian B. (2015). Investigation of microwave dryer effect on energy efficiency during drying of apple slices. J. Saudi Soc. Agric. Sci..

[4] Vega-Mercado H., Góngora-Nieto M.M., Barbosa-Cánovas G.V. (2001). Advances in dehydration of foods. J. Food Eng..

[5] Ratti C. (2001). Hot air and freeze-drying of high-value foods: A review. J. Food Eng..

[6] Nindo C.I., Tang J. (2007). Refractance window dehydration technology: A novel contact drying method. Dry. Technol. Int. J..

[7] Tontul I., Eroğlu E., Topuz A. (2018). Convective and refractance window drying of cornelian cherry pulp: Effect on physicochemical properties. J. Food Process Eng..

[8] Mahanti N.K., Chakraborty S.K., Sudhakar A., Verma D.K., Shankar S., Thakur M., Singh S., Tripathy S., Gupta A.K., Srivastav P.P. (2021). Refractance window^TM^-drying vs. other drying methods and effect of different process parameters on quality of foods: A comprehensive review of trends and technological developments. Future Foods.

[9] Shende D., Datta A.K. (2019). Refractance window drying of fruits and vegetables: A review. J. Sci. Food Agric..

[10] Bampi M., Domschke N.N., Schmidt F.C., Laurindo J.B. (2016). Influence of vacuum application, acid addition and partial replacement of NaCl by KCl on the mass transfer during salting of beef cuts. LWT Food Sci. Technol..

[11] González-Pérez J.E., Jiménez-González O., Ramírez-Corona N., Guerrero-Beltrán J.A., López-Malo A. (2022). Vacuum impregnation on apples with grape juice concentrate: Effects of pressure, processing time, and juice concentration. Innov. Food Sci. Emerg. Technol..

[12] Pasławska M., Stepien B., Nawirska-Olszanska A., Sala K. (2019). Studies on the effect of mass transfer in vacuum impregnation on the bioactive potential of apples. Molecules.

[13] Erihemu, Hironaka K., Oda Y., Koaze H. (2014). Iron enrichment of whole potato tuber by vacuum impregnation. LWT-Food Sci. Technol..

[14] Aamir M., Jittanit W. (2017). Ohmic heating treatment for gac aril oil extraction: Effects on extraction efficiency, physical properties and some bioactive compounds. Innov. Food Sci. Emerg. Technol..

[15] Kusnadi C., Sastry S.K. (2012). Effect of moderate electric fields on salt diffusion into vegetable tissue. J. Food Eng..

[16] Moreno J., Espinoza C., Simpson R., Petzold G., Nuñez H., Gianelli M.P. (2016). Application of ohmic heating/vacuum impregnation treatments and air drying to develop an apple snack enriched in folic acid. Innov. Food Sci. Emerg. Technol..

[17] Simpson R., Ramírez C., Birchmeier V., Almonacid A., Moreno J., Nuñez H., Jaques A. (2015). Diffusion mechanisms during the osmotic dehydration of Granny Smith apples subjected to a moderate electric field. J. Food Eng..

[18] Araya E., Nuñez H., Ramírez N., Jaques A., Simpson R., Escobar M., Escalona P., Vega-Castro O., Ramírez C. (2022). Exploring the potential acceleration of Granny Smith apple drying by pre-treatment with CO_2_ laser microperforation. Food Bioprocess Technol..

[19] Ferraz A.C.O., Mittal G.S., Bilanski W.K., Abdullah H.A. (2007). Mathematical modeling of laser based potato cutting and peeling. BioSystems.

[20] Figueroa C., Ramírez C., Núñez H., Jaques A., Simpson R. (2020). Application of vacuum impregnation and CO_2_-laser microperforations in the potential acceleration of the pork marinating process. Innov. Food Sci. Emerg. Technol..

[21] Fujimaru T., Ling Q., Morrissey M.T. (2012). Effects of carbon dioxide (CO_2_) laserperforation as skin pretreatment to improve sugar infusion process of frozen blueberries. J. Food Sci..

[22] Olivares J., Nuñez H., Ramírez C., Jaques A., Pinto M., Fuentes L., Almonacid S., Vega-Castro O., Simpson R. (2021). Application of moderate electric fields and CO_2_-laser microperforations for the acceleration of the salting process of Atlantic salmon (*Salmo salar*). Food Bioprod. Process..

[23] Teng X., Zhang M., Mujumdar A.S. (2021). Potential application of laser technology in food processing. Trends Food Sci. Technol..

[24] Veloso G., Simpson R., Núñez H., Ramírez C., Almonacid S., Jaques A. (2021). Exploring the potential acceleration of the osmotic dehydration process via pretreatment with CO_2_-laser microperforations. J. Food Eng..

[25] Silva-Vera W., Avendaño-Muñoz N., Nuñez H., Ramírez C., Almonacid S., Simpson R. (2020). CO_2_ laser drilling coupled with moderate electric fields for enhancement of the mass transfer phenomenon in a tomato (*Lycopersicon esculentum*) peeling process. J. Food Eng..

[26] Tanzi E.L., Lupton J.R., Alster T.S. (2003). Lasers in dermatology: Four decades of progress. J. Am. Acad. Dermatol..

[27] Chen F., Zhang M., Devahastin S., Yu D. (2021). Comparative evaluation of the properties of deep-frozen blueberries dried by vacuum infrared freeze drying with the use of CO_2_ laser perforation, ultrasound, and freezing–thawing as pretreatments. Food Bioprocess Technol..

[28] Ramírez C., Troncoso E., Muñoz J., Aguilera J.M. (2011). Microstructure analysis on pre-treated apple slices and its effect on water release during air drying. J. Food Eng..

[29] Hernández Y., Ramírez C., Moreno J., Núñez H., Vega O., Almonacid S., Pinto M., Fuentes L., Simpson R. (2020). Effect of refractance window on dehydration of osmotically pretreated apple slices: Color and texture evaluation. J. Food Process Eng..

[30] Nieto A.B., Vicente S., Hodara K., Castro M.A., Alzamora S.M. (2013). Osmotic dehydration of apple: Influence of sugar and water activity on tissue structure, rheological properties and water mobility. J. Food Eng..

[31] Moraga G., Talens P., Moraga M.J., Martínez-Navarrete N. (2011). Implication of water activity and glass transition on the mechanical and optical properties of freeze-dried apple and banana slices. J. Food Eng..

[32] AOAC (2016). Official Methods of Analysis of AOAC International.

[33] Sturm B., Nunez Vega A.M., Hofacker W.C. (2014). Influence of process control strategies on drying kinetics, colour and shrinkage of air dried apples. Appl. Therm. Eng..

[34] Galaz P., Valdenegro M., Ramírez C., Nuñez H., Almonacid S., Simpson R. (2017). Effect of drum drying temperature on drying kinetic and polyphenol contents in pomegranate peel. J. Food Eng..

[35] Rajoriya D., Shewale S.R., Hebbar H.U. (2019). Refractance window drying of apple slices: Mass transfer phenomena and quality parameters. Food Bioprocess Technol..

[36] Henríquez C., Almonacid S., Chiffelle I., Valenzuela T., Araya M., Cabezas L., Simpson R., Speisky H. (2010). Determination of antioxidant capacity, total phenolic content and mineral composition of different fruit tissue of five apple cultivars grown in Chile. Chil. J. Agric. Res..

[37] Franco S., Jaques A., Pinto M., Fardella M., Valencia P., Núñez H., Ramírez C., Simpson R. (2019). Dehydration of salmon (*Atlantic salmon)*, beef, and apple (Granny Smith) using refractance window^TM^: Effect on diffusion behavior, texture, and color changes. Innov. Food Sci. Emerg. Technol..

[38] Caparino O.A., Tang J., Nindo C.I., Sablani S.S., Powers J.R., Fellman J.K. (2012). Effect of drying methods on the physical properties and microstructures of mango (*Philippine* “*Carabao*” var.) powder. J. Food Eng..

[39] Puente L., Vega-Gálvez A., Ah-Hen K.S., Rodríguez A., Pasten A., Poblete J., Pardo-Orellana C., Muñoz M. (2020). Refractance window drying of goldenberry (*Physalis peruviana* L.) pulp: A comparison of quality characteristics with respect to other drying techniques. LWT-Food Sci. Technol..

[40] Calín-Sánchez Á., Lipan L., Cano-Lamadrid M., Kharaghani A., Masztalerz K., Carbonell-Barrachina Á.A., Figiel A. (2020). Comparison of traditional and novel drying techniques and its effect on quality of fruits, vegetables and aromatic herbs. Foods.

[41] Wojdyło A., Figiel A., Legua P., Lech K., Carbonell-Barrachina Á.A., Hernández F. (2016). Chemical composition, antioxidant capacity, and sensory quality of dried jujube fruits as affected by cultivar and drying method. Food Chem..

[42] Mitić S.S., Stojanović B.T., Stojković M.B., Mitić M.N., Pavlović J.L. (2013). Total phenolics, flavonoids and antioxidant activity of different apple cultivars. Bulg. Chem. Commun..

[43] Rajoriya D., Shewale S.R., Bhavya M.L., Hebbar H.U. (2020). Far infrared assisted refractance window drying of apple slices: Comparative study on flavour, nutrient retention and drying characteristics. Innov. Food Sci. Emerg. Technol..

[44] Rajoriya D., Bhavya M.L., Hebbar H.U. (2021). Impact of process parameters on drying behaviour, mass transfer and quality profile of refractance window dried banana puree. LWT-Food Sci. Technol..

[45] Moses J.A., Norton T., Alagusundaram K., Tiwari B.K. (2014). Novel drying techniques for the food industry. Food Eng. Rev..

[46] Zotarelli M.F., Carciofi B.A.M., Laurindo J.B. (2015). Effect of process variables on the drying rate of mango pulp by refractance window. Food Res. Int..

[47] Ortiz-Jerez M.J., Gulati T., Datta A.K., Ochoa-Martínez C.I. (2015). Quantitative understanding of refractance window^TM^ drying. Food Bioprod. Process..

[48] Rocha-Parra D.F., Lanari M.C., Zamora M.C., Chirife J. (2016). Influence of storage conditions on phenolic compounds stability, antioxidant capacity and colour of freeze-dried encapsulated red wine. LWT-Food Sci. Technol..

[49] Tonon R.V., Brabet C., Hubinger M.D. (2010). Anthocyanin stability and antioxidant activity of spray-dried açai (*Euterpe oleracea* Mart.) juice produced with different carrier agents. Food Res. Int..

[50] Henríquez C., Córdova A., Almonacid S., Saavedra J. (2014). Kinetic modeling of phenolic compound degradation during drum-drying of apple peel by-products. J. Food Eng..

[51] Baeghbali V., Niakousari M., Farahnaky A. (2016). Refractance window drying of pomegranate juice: Quality retention and energy efficiency. LWT-Food Sci. Technol..

[52] Nayak B., Berrios J.D.J., Powers J.R., Tang J. (2011). Effect of extrusion on the antioxidant capacity and color attributes of expanded extrudates prepared from purple potato and yellow pea flour mixes. J. Food Sci..

[53] Robertson G.L., Robertson G.L. (2010). Food packaging and shelf life. Food Packaging and Shelf Life.

[54] Sablani S.S. (2006). Drying of fruits and vegetables: Retention of nutritional/functional quality. Dry. Technol..

[55] Lavelli V., Vantaggi C. (2009). Rate of antioxidant degradation and color variations in dehydrated apples as related to water activity. J. Agric. Food Chem..

[56] Henríquez M., Almonacid S., Lutz M., Simpson R., Valdenegro M. (2013). Comparison of three drying processes to obtain an apple peel food ingredient. CYTA-J. Food.

[57] Bonazzi C., Dumoulin E. (2014). Quality changes in food materials as influenced by drying processes. Mod. Dry. Technol..

